# Marburg virus disease: the paradox of Nigeria’s preparedness and priority effects in co-epidemics

**DOI:** 10.1186/s42269-023-00987-1

**Published:** 2023-01-27

**Authors:** Rine Christopher Reuben, Sarah Adamma Abunike

**Affiliations:** 1 German Centre of Integrative Biodiversity Research (iDiv) Halle-Jena-Leipzig, Puschstraße 4, 04103 Leipzig, Germany; 2 Institute of Biology, Leipzig University, Puschstraße 4, 04103 Leipzig, Germany; 3 Department of Biological Science, Anchor University, Lagos, Nigeria; 4 Institute for Health and Equity, Medical College of Wisconsin, 8701 Watertown Plank Road, Milwaukee, WI 53226 USA

**Keywords:** Marburg virus disease, COVID-19, Public health, Co-epidemics, Pandemic

## Abstract

**Background:**

The recent outbreaks of Marburg virus disease (MVD) in Guinea and Ghana have become a major public health concern not only to the West African sub-region but a threat to global health.

**Main body of the abstract:**

Given the poorly elucidated ecological and epidemiological dynamics of the Marburg virus, it would be imprudent to preclude the possibility of another pandemic if urgent efforts are not put in place. However, the prior emergence and impact of COVID-19 and other co-occurring epidemics may add ‘noise’ to the epidemiological dynamics and public health interventions that may be required in the advent of a MVD outbreak in Nigeria.

**Short conclusion:**

Paying attention to the lessons learned from previous (and current) multiple epidemics including Avian Influenza, Yellow fever, Ebola virus disease, Monkeypox, Lassa fever, and COVID-19 could help avoid a potentially devastating public health catastrophe in Nigeria.

## Background

Marburg virus disease (MVD) is a highly contagious, fatal, and infectious disease caused by the Marburg virus (MARV), a member of the *Filoviridae* family, and closely related to the widely known Ebola virus (EBOV) (Shi et al. 2018; Kuhn et al. 2019; WHO, 2021a). Similar to other filoviruses, MARV causes acute and lethal haemorrhagic fever in humans (and nonhuman primates (NHPs)) with high case fatality rates (CFR) ranging from 24 to 90% (Languon et al. 2019; Shifflett et al. 2019; WHO 2021). MARV transmission occurs primarily through direct contact with the natural reservoir host (strongly believed to be Egyptian fruit bats, *Roussettus aegypticus*) or their excreta (Changula et al. 2014; Amman et al. 2015; Pawęska, et al. 2018; Kajihara et al. 2019). Secondary transmission occurs through contact with blood or body secretions/excretions of infected humans, animals, or with materials and surfaces (e.g. bedding, clothing) contaminated with these secretions, and also, nosocomially, mostly through parenteral or unprotected exposure (Sanchez et al. 2007; Mehedi et al. 2011a, b; Kortepeter et al. 2020WHO 2021a).

Although detailed pathophysiological mechanisms and processes characterizing MVD largely remain elusive, the MVD incubation period ranges from 2 to 21 days and initially presents with influenza-like symptoms, high fever, myalgia and fatigue, dysphagia, dyspnea, edema, which further progresses to neurological symptoms including encephalitis, confusion, delirium, irritability, and aggression. During the later phase of the disease, patients may develop several hemorrhagic manifestations (e.g. mucosal bleeding, petechiae, visceral hemorrhagic effusions, melena, bloody and severe diarrhea, hematemesis, and uncontrolled leakage from venipuncture sites, which finally lead to multiple organ failure, shock, and death (Borchert et al. 2002; Bausch et al. 2006; Colebunders et al. 2007; Sanchez et al. 2007; Mehedi et al. 2011a, b; Okonji et al. 2022).

Since the first reported outbreak of MVD in the 1960s in Marburg, Germany, and also in Belgrade, Serbia, where laboratorians got infected while working with *Cercopithecus aethiops* (African green monkeys) imported from Uganda, several unrelated and sporadic outbreaks have been documented (Smith and Simpson 1967; Martini et al. 1968; Henderson et al. 1971; Martini 1971; Slenczka and Klenk 2007; WHO 2021b). Though MVD emerged in Europe, however, majority of the outbreaks mostly occur in Africa. Over the years, outbreaks and sporadic cases of MVD have been repeatedly reported in Angola, Kenya, the Democratic Republic of Congo, South Africa, Uganda, Zimbabwe, and recently the Republic of Guinea, and Ghana respectively (WHO 2021b; WHO 2022a). Whereas some outbreaks consist of a small cluster of cases (as in South Africa/Zimbabwe in 1975) (Conrad et al., 1978; WHO, 2021b; ECDC, 2022), other results in high morbidity and mortality. For example, in the 2005 MVD outbreaks in Angola, 374 cases were confirmed with 329 mortality (88% CFR) (Towner et al. 2006; WHO 2021b; ECDC 2022). Four outbreaks have been reported in Uganda in 2007, 2012, 2014, and 2017 with CFR between 27 and 100%. Between 1998 and 2000, 154 cases were reported in Congo with 128 mortality (83% CFR) (Leroy et al. 2011; WHO 2021b; ECDC 2022). Furthermore, fatal outbreaks of MVD were previously reported in the USA as well as the Netherlands in 2008. Both cases were reportedly imported from visits to a cave in western Uganda (Mehedi et al. 2011a, b; Brauburger et al. 2012).

For more than five (5) decades since the emergence of MARV, no countermeasures including prophylactic, therapeutic, and/or vaccines have been successfully developed and approved worldwide. Individuals infected with MARV are often isolated, closely monitored for clinical manifestations, and given supportive care. However, three Marburg vaccine candidates (MARV DNA, MVA-BN-Filo, and cAd3) are in Phase I clinical trials with one, MVA-BN-Filo scheduled for the second phase of the trial (Reynolds and Marzi, 2017; Kortepeter et al. 2020). Furthermore, the recent recurrent West African Ebola virus disease (EBV) epidemics have increased joint efforts focused on developing vaccines and therapeutic countermeasures against EVD as well as MVD (Olejnik et al., 2019).

## Main text

### The current MDV epidemic and associated concerns

In 2021, the first West African outbreak of MVD was reported in Guinea with 100% CFR. The sudden emergence of MVD in West Africa was initially surprising, however, ecological niche modeling has previously demonstrated that the West African sub-region is one of the regions with similar ecological features to other MARV endemic areas and is at high risk of MVD (Peterson et al. 2006). Although the cause, origin/source, and reason for the first MVD outbreak in West Africa still remain unknown, the simultaneous emergence of MVD amidst the current COVID-19 pandemic raises enormous public health concerns not only for the West African sub-region but the world over.

On the 17th of July 2022, the World Health Organization confirm the latest MVD outbreak in Ghana (WHO, 2022a). This is the second MVD outbreak in the West African subregion following the initial 2021 outbreak in Guinea. The WHO has confirmed four unrelated MVD cases reported in the Adansi North District, Ashanti region and Sawla-Tuna-Kalba District, Savannah Region with 3 mortalities (75% CFR) (CDC 2022). Through contact tracing, follow-up, and testing the Ghana Health Service and the WHO are conducting case investigations, identifying sources of transmissions, reinforcing surveillance, and also, educating communities and local populations about the dangers and risks of MVD (CDC 2022). Given the poorly elucidated ecological and evolutionary dynamics, and epidemiology of MAVR, Nigeria’s proximity to Ghana, and the high human traffic (which is poorly and seldom regulated) between both countries, the Nigerian population is at a very high risk of MVD. Furthermore, Nigeria’s poor disease surveillance and reporting could unduly expose its population to MVD due to its (often) inadequate coordination and preparedness for epidemics.

Unfortunately, Africa has within the last decade witnessed a 63% increase in zoonotic disease of which 70% are due to deadly emerging viruses (WHO 2022b). This has created a huge burden not only on African healthcare systems but on the overall quality of life (Reuben et al. 2020; Nnaji et al. 2021). Recent studies showed how Nigeria's (and the majority of African states) clinical and public health have been adversely impacted by the simultaneous occurrence of epidemics amidst multiple endemic diseases (Nnaji et al. 2021; Uwishema et al. 2021).

Long before the emergence of COVID-19, Nigeria has been battling with endemic and recurrent outbreaks of Lassa fever (LF), a zoonotic and viral haemorrhagic fever (Usuwa et al. 2020). Though deadlier than COVID-19, the emergence of COVID-19 has seldom masked LF surveillance, reporting, and interventions in Nigeria (Reuben et al. 2020; 2021, Musa et al. 2022). Various other infectious diseases of epidemic proportion circulating in different (rural) communities have either been underreported, underrated, and/or not given the needed attention, and in most cases undetected. Recent Nigeria Centre for Disease Control (NCDC) weekly epidemiological reports show increasing cases and outbreaks of different fatal and life-threatening infectious diseases of epidemic (and pandemic) proportions including Lassa fever, Avian Influenza, Yellow fever, Rubella, Measles, Meningitis and cholera (NCDC, 2022a). Also, the recent outbreaks and soaring cases of Monkeypox in Nigeria (with about a thousand cases reported in 30 states) have further aggravated the already existing public health problems (NCDC 2022b).

The COVID-19 pandemic caused multiple healthcare-associated disruptions in several (developing) countries including Nigeria, which has contributed in the recent rising cases of multiple diseases (Abbas et al. 2021; Hasan et al. 2021; Khan et al. 2021; Mohan et al. 2021; Nnaji et al. 2021; Yousaf et al. 2021). In Nigeria for instance, the COVID-19 pandemic negatively affected surveillance, testing, reporting, and treatment for other endemic (and emerging) infectious diseases (Aborode et al. 2021; Saalim et al. 2021). The unprecedented measures adopted and implemented by the Nigerian government in mitigating the pandemic increased the difficulty in accessing healthcare services. Furthermore, Nigeria’s weak healthcare systems were overburdened thus, negatively impacting patients' care and management.

### Priority effects in co-epidemics dynamics and mitigation

The epidemiological dynamics of the emergence and spread of infectious diseases are complex and mostly influenced by changes in multiple biological, demographic, environmental, and socioeconomic factors (Morse 2004; Chan et al. 2013). Recently, Nigeria has been witnessing an upsurge of co-epidemics with significant commonalities in both clinical presentation and public health response. Priority effects, though widely used in ecology refer to the negative or positive impacts species will have on one another due to the order and/or timing of their arrival in a community (Sprockett et al. 2018; Weidlich et al. 2021; Debray et al. 2022). Prior arrival or emergence influences succession when species with prior arrival history alter environmental conditions or resources which significantly affects species that arrive later, consequentially impacting their growth and survival in the community.

The co-occurrence of epidemics can decrease or increase one another’s epidemic severity and trajectories by interacting at the host population (or individual scale) (Clay et al. 2018, 2020). Thus, understanding infectious disease dynamics (even before emergence in a population) is imperative for timely public health interventions and resource allocation. For instance, recent reports showed how the recurrent Lassa fever outbreaks in Nigeria impacted public health interventions towards the emergence of COVID-19 (Reuben et al. 2020; Uwishema et al. 2021; Musa et al. 2022). Similarly, we opine that the prior emergence and prolonged impact of COVID-19 may be important and also add ‘noise’ to epidemiological dynamics and interventions that may be required in case of a MVD outbreak in Nigeria. Because the control and/or prevention of epidemics is essentially dependent on limited sources (manpower, facilities, and funds), effectively mitigating multiple epidemics within a population can depend on the order and/or timing of their emergence, severity, or epidemiology. Furthermore, Nigeria’s capacity to effectively manage infectious disease outbreaks remains suboptimal, largely due to poor surveillance and record management, inadequate diagnosis, weakened infection prevention and control (IPC) measures, and insufficient health funding amongst others.

The back-to-back recurrent outbreaks of MVD in West African states (first in Guinea and currently, Ghana) within 12 months interval is worrisome, and a great threat to sub-regional public health. Since Nigeria occupies the largest land area with about ½ of the entire West African population (UN, 2022), there is a need for robust preparedness in the event of a MVD outbreak. The major complication in the first West African (Guinea) and the current (Ghana) outbreaks of MVD is the unknown source(s) of infection in the index cases. Other public health challenges were obvious gaps in response capacity including giving inadequate attention as required, poor contact tracing and missing of high-risk contacts, and inadequate IPC measures in communities and healthcare facilities (WHO 2021c; Okonji et al. 2022). The proximity (due to shared land boundaries) among West African states usually allows easy, frequent, and unchecked cross-border movements of people (and goods) which could easily contribute to the further spread of disease including MVD to neighboring states. Apart from being overburdened with multiple epidemics, inadequate access to (primary) healthcare services, insufficient public health infrastructure and funding, and poor IPC measures have always been major impediments in the management of epidemics in Nigeria (Buseh and Stevens 2015; Nnaji et al. 2021; Musa et al. 2022). Furthermore, the weakness of disease surveillance and diagnosis in Nigeria (and the West Africa sub-region) is another major concern that could facilitate infectious disease outbreaks with devastating consequences (Buesen et al. 2015; Aborode et al. 2021; Nnaji et al. 2021; WHO, 2021b).

### What should Nigeria do?

The COVID-19 pandemic has continued to adversely impact Nigeria, making any MVD epidemic to further exacerbate the already overburdened healthcare systems. Howbeit, giving due diligence to the lessons learned from previous (and current) multiple epidemics including Avian Influenza, Yellow fever, Ebola virus disease, Monkeypox, Lassa Fever, and currently COVID-19 could help avoid a potentially devastating public health catastrophe. The entire country especially at the sub-national levels should ensure early readiness and seriousness of their healthcare systems to massively tackle suspected MVD cases in a coordinated, timely, and efficient matter. Disease surveillance networks should be intensified and widened to determine and track individuals and communities at risk (e.g. communities at the borders and fringes) and implement appropriate IPC measures to protect them as well as their contacts. To avoid misconceptions and stigmatization which could hamper contact tracing (as in the case of Lassa fever, Ebola virus disease, and COVID-19), urgent and massive MVD-related public health enlightenment campaigns in all the federating Nigerian states, local government areas, and districts are necessary for knowledge and optimistic attitudes in the advent of a MDV outbreak.

The WHO concerted One Health approach should be implemented across the animal-human–environment continuum, hence fostering multi-sectoral collaboration and synergy. Early preparedness and readiness of both the local and state governments' healthcare systems are crucial to tackle MVD outbreak in a concerted and coordinated timely manner. Accordingly, it is essential to enhance training and/or retraining of frontline healthcare workers on (1) proper and timely MVD diagnosis, (2) MDV care and management, and (3) logistics, in case of a MVD outbreak. The establishment of skilled, free, and easily accessible community clinics for massive screening (or sample collection) to help with the early detection of MDV is highly recommended. In addition, the NCDC in conjunction with the Federal Ministry of Health should set up national guidelines for case definitions of MDV, care and management, and IPC measure through national policies.

Nigeria’s increasing number of internet and social media users (currently 117.87 and 32.9 million, respectively, based on 2022 data) (Sasu 2022a; b), can both be armed and used to increase public enlightenment, knowledge, and practices regarding MDV. Accurate medical information about MDV can be used to attenuate potential MDV-associated misconceptions through multiple flag-based systems across various social media platforms to avert any possible spread of potentially offensive MDV-related content which could be viral.

Apart from the healthcare settings, most infectious diseases are largely transmitted at the household level (Reichler et al. 2018; Caleo et al. 2018; Beale et al. 2020; Makinde et al. 2021). Therefore, certain IPC measures successfully implemented at the household level during the repeated outbreaks of emerging diseases (e.g. Ebola virus disease) in West African countries could be replicated to mitigate eventual co-epidemics of MVD with either Lassa fever, COVID-19, Avian Influenza, Yellow fever, Ebola virus disease, Monkeypox, or any other deadly infectious disease(s). At the household level, routine social mobilization programmes aimed at preventing infection should be targeted at children and women to ensure they comprehend basic IPC measures. Public health campaign messages/strategies should be in clear, simple, and easy-to-understand language that required the cooperation and involvement of children, women, and other household members. This could include the constant provision of soap and water for hand washing, and the procurement of sanitizers and other household hygiene kits. Furthermore, within households, contacts who provided care to the index case, or had physical contact with the case or other contaminated household materials should be considered high risk. More intensive house-to-house monitoring of high-risk contacts as well as rapid diagnosis of suspected cases would significantly minimize household (and community) transmission.

## Conclusions

The recurrent outbreaks of MDV in West Africa have become a major public health concern not only to Nigeria but the entire sub-region, and a threat to global health. Though the source(s) of the West African outbreaks of MDV remain unknown, it would be imprudent to preclude the possibility of another pandemic if urgent efforts are not put in place. To this end, concerted, timely, and urgent preparedness of West African states including Nigeria against the outbreak of MDV becomes imperative. Proper surveillance across Nigeria especially in suspected high-risk areas should be intensified. Healthcare personnel should be (re)trained and routinely updated on the co-occurrence of multiple outbreaks of deadly viruses and the high potential of the emergence of MARV. For every possible clinical manifestation of MDV, early isolation, laboratory diagnosis, and contact tracing should be mandated. Concerted steps should be taken by the government and Non-governmental organizations to engage the medical community, epidemiologists, and researchers in community-based public health campaigns, planning, policy-making, and data collection and dissemination in the event of an outbreak. Introspecting the current distressing situation of Nigeria’s healthcare systems by COVID-19 (and other co-epidemics), another outbreak by a deadly virus especially MAVR could result in a national healthcare crisis that may not be quickly mitigated. Therefore, a well-guided beforehand protocol for mitigating and/or minimizing MVD outbreaks in Nigeria is necessary.

## Data Availability

Not applicable.

## Abbreviations

WHO EBOV: Ebola virus
NHPs: Nonhuman primates
CFR: Case fatality rates
ECDC: European centre for disease prevention and control
IPC: Infection, prevention and control. world health organization
CDC: Centers for disease control and prevention
NCDC: Nigeria center for disease control
MVD: Marburg virus disease
MAVR: Marburg virus

## References

[CR1] Abbas KS, Kaml MBS, Metry MAF (2021). Hepatitis C liver disease during COVID-19 pandemic in Egypt: challenges and way forward. Ethics Med Public Heal.

[CR2] Aborode AT, Hasan MM, Jain S (2021). Impact of poor disease surveillance system on COVID-19 response in Africa: time to rethink and rebuilt. Clin Epidemiol Glob Heal.

[CR3] Amman BR, Jones MEB, Sealy TK, Uebelhoer LS, Schuh AJ, Bird BH (2015). Oral shedding of Marburg virus in experimentally infected Egyptian fruit bats (*Rousettus aegyptiacus*). J Wildl Dis.

[CR4] Bausch DG, Nichol ST, Muyembe-Tamfum JJ (2006). Marburg hemorrhagic fever associated with multiple genetic lineages of virus. N Engl J Med.

[CR5] Beale S, Lewer D, Aldridge RW (2020). Household transmission of seasonal coronavirus infections: results from the Flu watch cohort study. Wellcome Open Res.

[CR6] Borchert M, Muyembe-Tamfum JJ, Colebunders R, Libande M, Sabue M, Van Der Stuyft P (2002). Short communication: a cluster of Marburg virus disease involving an infant. Trop Med Int Health.

[CR7] Brauburger K, Hume AJ, Mühlberger E, Olejnik J (2012). Forty-five years of Marburg virus research. Viruses.

[CR8] Buesen AG, Stevens PE, Bromberg M, Kelber ST (2015). The Ebola epidemic in West Africa: challenges, opportunities, and policy priority areas. Nurs Outlook.

[CR9] Buseh AG, Stevens PE (2015). The Ebola epidemic in West Africa: challenges, opportunities, and policy priority areas. Nurs Outlook.

[CR10] Caleo G, Duncombe J, Jephcott F (2018). The factors affecting household transmission dynamics and community compliance with Ebola control measures: a mixed-methods study in a rural village in Sierra Leone. BMC Public Health.

[CR11] Centers for Disease control and prevention CDC, (2022). Marburg in Ghana. https://wwwnc.cdc.gov/travel/notices/watch/marburg-ghana (Accessed 5 Aug 2022)

[CR12] Chan EH, Scales DA, Brewer TF, Madoff LC, Pollack MP, Hoen AG (2013). Forecasting high-priority infectious disease surveillance regions: a socioeconomic model priority effects, infectious disease dynamics, and Nigeria’s preparedness. Clin Infect Dis.

[CR13] Changula K, Kajihara M, Mweene AS, Takada A (2014). Ebola and Marburg virus diseases in Africa: increased risk of outbreaks in previously unaffected areas?. Microbiol Immunol.

[CR14] Clay PA, Dhir K, Rudolf VHW, Duffy MA (2018). Within-host priority effects systematically alter pathogen coexistence. Am Nat.

[CR15] Clay PA, Duffy MA, Rudolf VHW (2020). Within-host priority effects and epidemic timing determine outbreak severity in co-infected populations. Proc R Soc B.

[CR16] Colebunders R, Tshomba A, Van Kerkhove MD (2007). Marburg hemorrhagic fever in Durba and Watsa, democratic republic of the Congo: clinical documentation, features of illness, and treatment. J Infect Dis.

[CR17] Conrad JL, Isaacson M, Smith EB (1978). Epidemiologic investigation of Marburg virus disease, Southern Africa, 1975. Am J Trop Med Hyg.

[CR18] Debray R, Herbert RA, Jaffe AL (2022). Priority effects in microbiome assembly. Nat Rev Microbiol.

[CR19] European centre for disease prevention and control ECDC (2022). Marburg virus disease outbreaks in Africa as of 31 May 2022. https://www.ecdc.europa.eu/en/publications-data/marburg-virus-disease-outbreaks-africa-31-may-2022 (Accessed 29 July 2022)

[CR20] Hasan MM, Costa AC, dos S, Xenophontos E, (2021). Lassa fever and COVID-19 in Africa: a double crisis on the fragile health system. J Med Virol.

[CR21] Henderson BE, Kissling RE, Williams MC, Kafuko GW, Martin M, Martini GA, Siegert R (1971). Epidemiological studies in uganda relating to the “Marburg” Agent. Marburg Virus Disease.

[CR22] Kajihara M, Hang’ombe BM, Changula K, Harima H, Isono M, Okuya K, Takada, A. (2019). Marburgvirus in Egyptian fruit bats. Zambia Emerg Infect Dis.

[CR23] Khan FMA, Kazmi Z, Hasan MM (2021). Resurgence of tuberculosis amid COVID-19 in Peru: associated risk factors and recommendations. Int J Health Plann Manag.

[CR24] Kortepeter MG, Kerry DE, Shenoy TJC (2020). Marburg virus disease: a summary for clinicians. Intl J Infect Dis.

[CR25] Kuhn JH, Adachi T, Adhikari NKJ, Arribas JR, Bah IE, Bausch DG (2019). New filovirus disease classification and nomenclature. Nat Rev Microbiol.

[CR26] Languon S, Quaye O (2019). Filovirus disease outbreaks: a chronological overview. Virology (auckl).

[CR27] Leroyab EM, Gonzalez JP, Baize S (2011). Ebola and Marburg haemorrhagic fever viruses: major scientific advances, but a relatively minor public health threat for Africa. Clin Microbiol Infect.

[CR28] Makinde OA, Akinyemi JO, Ntoimo LF (2021). Risk assessment for COVID-19 transmission at household level in sub-Saharan Africa: evidence from DHS. Genus.

[CR29] Martini GA (1971) Marburg virus disease. Clinical syndrome. In: Martini GA, Siegert R, eds. Marburg Virus Disease. Springer, Berlin., Heidelberg, pp 1–9 10.1007/978-3-662-01593-3_1

[CR30] Martini GA, Knauff HG, Schmidt HA, Mayer G, Baltzer G (1968). [On the hitherto unknown, in monkeys originating infectious disease: Marburg virus disease] Dtsch Med. Wochenschr.

[CR31] Mehedi M, Allison G, Heinz F, Hideki E (2011). clinical aspects of Marburg hemorrhagic fever. Future Virol.

[CR32] Mehedi M, Groseth A, Feldmann H, Ebihara H (2011). Clinical aspects of Marburg hemorrhagic fever. Future Virol.

[CR33] Mohan A, Temitope RA, Çavdaro ˘glu S, (2021). Measles returns to the democratic republic of Congo: a new predicament amid the COVID-19 crisis. J Med Virol.

[CR34] Morse SS (2004). Factors and determinants of disease emergence. Rev Sci Tech.

[CR35] Musa SS, Zhao S, Abdullahi ZU, Habib AG, He D (2022). COVID-19 and lassa fever in Nigeria: a deadly alliance?. Int J Infect Dis.

[CR36] Nigeria centre for disease control (NCDC) (2022a). Disease situation reports. https://ncdc.gov.ng/diseases/sitreps (Accessed Sep 17, 2022a).

[CR37] Nigeria centre for disease control (NCDC) (2022b). An update of Monkeypox outbreak in Nigeria. https://ncdc.gov.ng/diseases/sitreps/?cat=8&name=An%20Update%20of%20Monkeypox%20Outbreak%20in%20Nigeria (Accessed Sep 17, 2022b).

[CR38] Nnaji ND, Onyeaka H, Reuben RC (2021). The deuce-ace of lassa fever, Ebola virus disease and COVID-19 simultaneous infections and epidemics in West Africa: clinical and public health implications. Trop Med Health.

[CR39] Okonji OC, Okonji EF, Mohanan P, Babar MS, Saleem A, Khawaja UA (2022). Marburg virus disease outbreak amidst COVID-19 in the republic of Guinea: a point of contention for the fragile health system?. Clinic Epidemiol Global Health.

[CR40] Pawęska JT, Jansen van Vuren P, Kemp A, Storm N, Grobbelaar AA (2018). Marburg virus infection in Egyptian Rousette bats, South Africa, 2013–2014. Emerg Infect Dis.

[CR41] Peterson AT, Lash RR, Carroll DS, Johnson KM (2006). Geographic potential for outbreaks of Marburg hemorrhagic fever. Am J Trop Med Hyg.

[CR42] Reichler MR, Bangura J, Bruden D (2018). Household transmission of Ebola Virus: risks and preventive factors, freetown, Sierra Leone, 2015. J Infect Dis.

[CR43] Reuben RC, Danladi MM, Pennap GR (2020). Is the COVID-19 pandemic masking the deadlier Lassa fever epidemic in Nigeria?. J Clin Virol.

[CR44] Reuben RC, Gyar SD, Makut MD, Adoga MP (2021). Co-epidemics: have measures against COVID-19 helped to reduce Lassa fever cases in Nigeria?. New Microbes New Infect.

[CR45] Reynolds P, Marzi A (2017). Ebola and Marburg virus vaccines. Virus Genes.

[CR46] Saalim K, Sakyi KS, Fatema-Tuz-Zohra, (2021). Reported health and social consequences of the COVID-19 pandemic on vulnerable populations and implemented solutions in six West African countries: a media content analysis. PLoS ONE.

[CR47] Sasu DS (2022a). Internet users in Nigeria 2018–2022a, with forecasts up until 2027. https://www.statista.com/statistics/183849/internet-users-nigeria/ (Accessed Sep 17, 2022a).

[CR48] Sasu DS (2022b). Percentage change in number of social media users in Nigeria 2015–2022b. https://www.statista.com/statistics/1324463/growth-rate-of-number-of-social-media-users-in-nigeria/ (Accessed Sep 17, 2022b).

[CR49] Sanchez A, Geisbert T, Feldmann H, Knipe D (2007). (2007). *Filoviridae* – Marburg and ebola viruses. Fields Virology.

[CR50] Shi M, Lin XD, Chen X, Tian JH, Chen LJ, Li K (2018). The evolutionary history of vertebrate RNA viruses. Nature.

[CR51] Shifflett K, Marzi A (2019). Marburg virus pathogenesis – differences and similarities in humans and animal models. Virol J.

[CR52] Slenczka W, Klenk HD (2007). Forty years of Marburg virus. J Infect Diseases.

[CR53] Smith CEG, Simpson DIH, Bowen ETW (1967). Fatal human disease from vervet monkeys. Lancet.

[CR54] Sprockett D, Fukami T, Relman DA (2018). Role of priority effects in the early-life assembly of the gut microbiota. Nature Rev Gastroenterol Hepatol.

[CR55] Towner JS, Khristova ML, Sealy TK (2006). Marburg virus genomics and association with a large hemorrhagic fever outbreak in Angola. J Virol.

[CR56] United Nations (UN) (2022). World population prospects. https://population.un.org/wpp/ (Accessed Sep 17, 2022).

[CR57] Usuwa IS, Akpa CO, Umeokonkwo CD (2020). Knowledge and risk perception towards Lassa fever infection among residents of affected communities in Ebonyi State, Nigeria: implications for risk communication. BMC Public Health.

[CR58] Uwishema O, Baha AA, Alshareif MYE, Yousif MEA, Omer ALR, Sablay RT, Zahabioun A, Mwazighe RM, Onyeaka H (2021). Lassa fever amidst the COVID-19 pandemic in Africa: a rising concern, efforts, challenges, and future recommendations. J Med Virol.

[CR59] Weidlich EWA, Nelson CR, Maron JL, Callaway RM, Delory BM, Temperton VM (2021). Priority effects and ecological restoration. Restoration Ecol.

[CR60] World Health Organisation (WHO) (2021a). Marburg virus disease - Guinea; 2021a. https://www.who.int/emergencies/disease-outbreak-news/item/2021a-Don331 (Accessed Sep 7 2022)

[CR61] World Health Organisation (WHOb) (2021b). Marburg virus disease; 2021. https://www.who.int/news-room/fact-sheets/detail/marburg-virus-disease (Accessed Sep 7, 2022)

[CR62] World Health Organisation (WHO) (2021c). Weekly bulletins on outbreaks and other emergencies | WHO | regional office for Africa. https://www.afro.who.int/health-topics/disease-outbreaks/outbreaks-and-other-emergencies-updates (Accessed Sep 4, 2022)

[CR63] World Health Organization (WHO) (2022a). Marburg virus Ghana https://www.who.int/emergencies/disease-outbreak-news/item/2022a-DON402 (Accessed July 28, 2022a)

[CR64] World Health Organization (WHO) (2022b). In Africa, 63% jump in diseases spread from animals to people seen in last decade. https://www.afro.who.int/news/africa-63-jump-diseases-spread-animals-people-seen-last-decade (Accessed Sep 17, 2022)

[CR65] Yousaf A, Khan FMA, Hasan MM, Ullah I, Bardhan M (2021). Dengue, measles, and COVID-19: a threefold challenge to public health security in Pakistan. Ethics, Med Public Heal.

